# Supplementary material for: Simultaneous Spray Drying for Combination Dry Powder Inhaler Formulations
Source: Pharmaceutics. 2022 May 26;14(6):1130. doi: 10.3390/pharmaceutics14061130 (PMC9227944; doi:10.3390/pharmaceutics14061130)
Supplement: Supplementary file 1 [file pharmaceutics-14-01130-s001.zip › pharmaceutics-1726291-supplementary.pdf]

Supplementary Information for Shepard et al

Formulation and solvent selection

Formulation development for spray-dried bevacizumab (BEV) was performed in a previous publication (1). In summary, the BEV spray solution used for all formulations in this study consisted of 10 mg/mL total solids in 1mM pH 6.3 phosphate buffer. BEV, a protein, is only chemically stable in aqueous buffers and therefore cannot be spray dried from organic solvents.

As discussed in our previous publication [Shepard et al], L-leucine is a preferred excipient which improves the aerosol properties of inhaled powders. Therefore, all formulations discussed here contain L-leucine. For the small molecule spray solutions, an appropriate solvent was selected, prioritizing three attributes: API solubility of at least ~2 mg/mL, L-leucine solubility of at least ~2 mg/mL, and acceptable volatility for spray drying. Cisplatin (CP) has limited solubility in water of ~2.5 mg/mL (as reported by the manufacturer) and is insoluble in alcohols. As L-leucine is insoluble in most volatile solvents besides water and alcohols, water was chosen as CP's spray solvent. CP formulations were screened with 4:1, 2:1 and 1:1 ratios of trehalose to CP, plus 20% L-leucine. In all cases, PXRD and DSC indicated the presence of crystalline CP as well as an amorphous CP/trehalose mixed phase. Since the spray drying throughput was limited by the CP solubility, the lowest active loading was chosen for further study: 10/70/20 CP/trehalose/L-leucine.

ERL is insoluble in water at neutral pH (< 0.1 mg/mL). Previous work in our group demonstrated ERL is fairly soluble in methanol. After screening ERL and L-leucine's solubility in a series of methanol/water mixtures, 90/10 methanol/water by weight was selected as the solvent for spray drying. At this composition, L-leucine's solubility is 2.5 mg/mL, and ERL's is 25 mg/mL. Formulation screening sprays were performed with ERL and combinations of L-leucine and trehalose as excipients. PXRD demonstrated that ERL was crystalline when spray dried with or without trehalose. Likely due to ERL's low glass transition temperature (~37°C), the API could not be trapped in a co-amorphous phase with the trehalose. As a result, a formulation of 80/20 ERL/L-leucine was selected for combination spray studies.

PTX is also sparingly soluble in water at neutral pH. The manufacturer reported acceptable solubility in ethanol, so the solubility of PTX and L-leucine was screening in a series of ethanol/water mixtures. 80/20 ethanol/water by weight was selected, where L-leucine's solubility is 1.7 mg/mL and PTX's solubility is 9 mg/mL. Formulation screening sprays were performed with PTX and combinations of L-leucine and trehalose as excipients. PXRD and DSC screening showed that spray dried PTX was amorphous regardless of the presence of trehalose. PTX's glass transition temperature is high, with an onset temperature of 129°C, leading to good stability in the amorphous state. A formulation of 80/20 PTX/L-leucine was selected for combination spray studies.

Aerodynamic Particle Size:

In the main text, summary statistics for the powders' aerodynamic particle size was reported. In Figure SI A, the full particle size distribution is shown for three formulations.

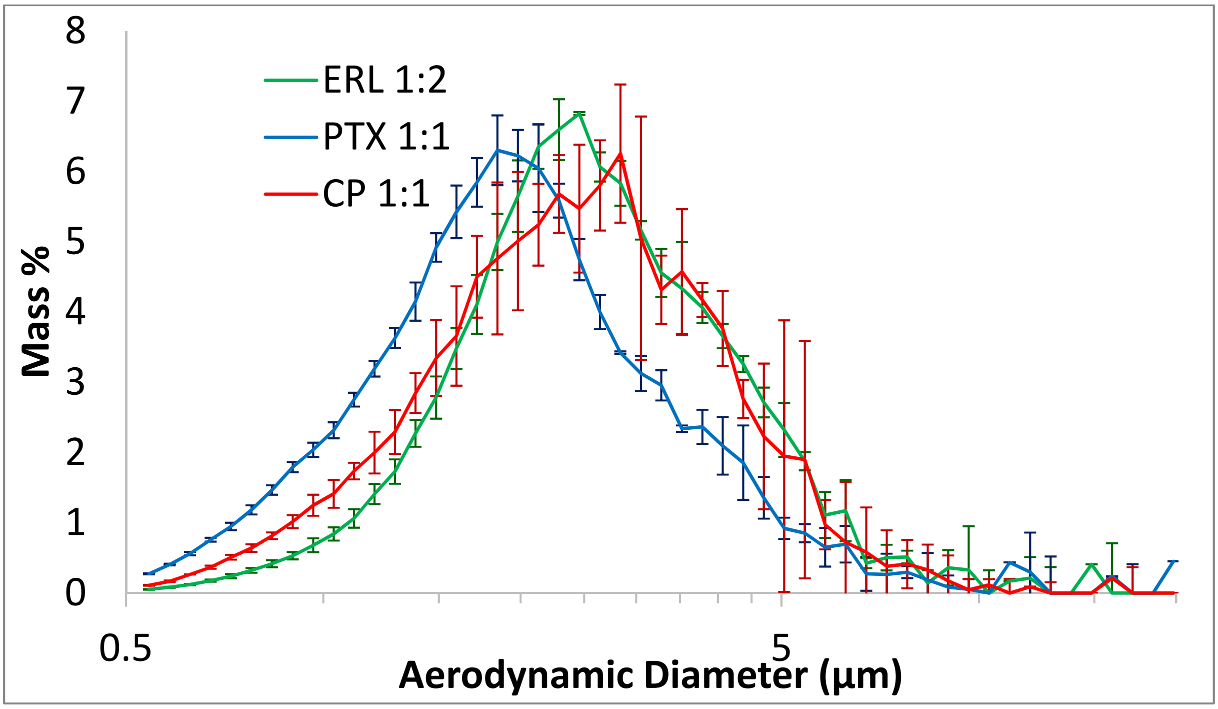

SI Figure A, Aerodynamic particle size distribution for ERL 1:2, PTX 1:1 and CP 1:1 formulations.

PXRD

X-Ray Diffraction was used to detect the presence of crystalline material in the simul-sprayed powders. Based on previous work, the BEV formulation has known peaks associated with crystalline L-leucine. For clarity, this data is reproduced in SI Figure B. For PTX 1:5, 1:2, 1:1, and 2:1, all formulations show the signature crystalline L-leucine peaks and an amorphous halo in the background, similarly to the Pac mono formulation. None of the peaks of crystalline PTX are found, suggesting that the powders consist of two phases: crystalline L-leucine and amorphous PTX.

For the ERL 1:1 and 1:2 formulations, the signature peaks of both ERL API and L-leucine are present in the ERL formulation, with a small amorphous halo observed (SI Figure C). In the ERL 1:1 trace, The L-leucine peaks are more prominent than the ERL peaks, and an amorphous halo is present, likely due to the BEV/trehalose amorphous phase. This indicates that the ERL is predominantly crystalline, but may have small amounts of amorphous material.

PXRD traces for CP 1:1, 2:1, CP mono and as-received CP API are overlayed in SI Figure D. None of the characteristic peaks of the CP API are present in spray-dried material, suggesting that the API is amorphous inside the trehalose matrix. However, the low active loading of these formulations could lead to crystallinity below the limit of detection for this instrument.

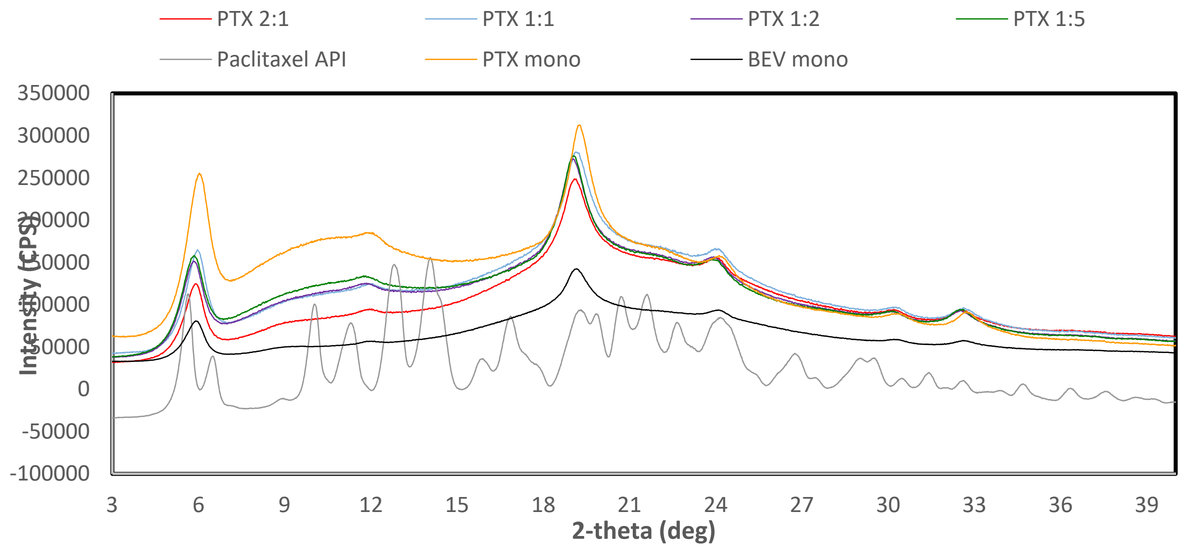

SI Figure B, PXRD of PTX 1:5, 1:2, 1:1, 2:1 formulations and controls.

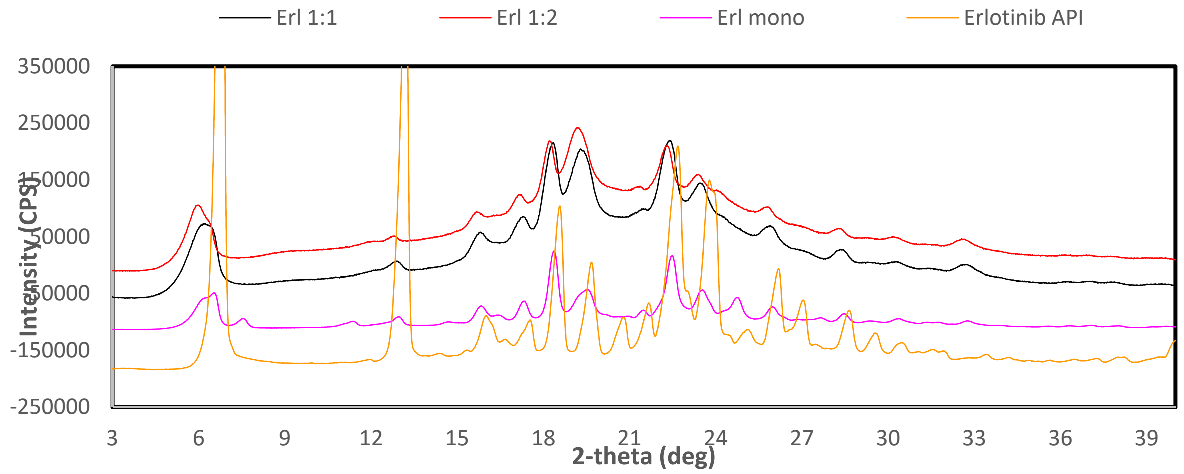

SI Figure C, PXRD of ERL 1:2, 1:1 formulations and controls.

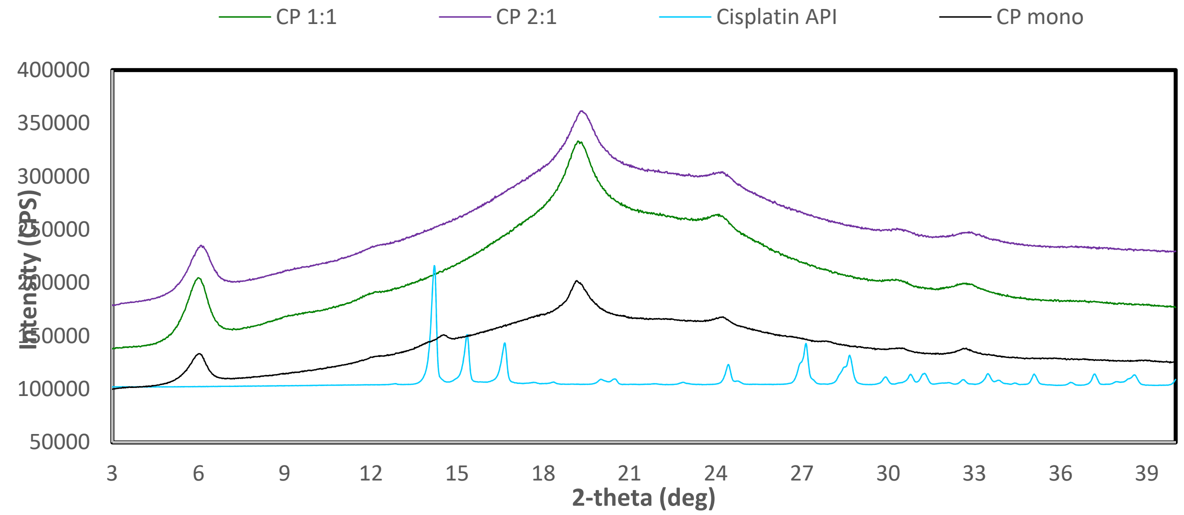

SI Figure D, PXRD of CP 1:1, 2:1 formulations and controls.

Thermal analysis

Thermal transitions of simul-sprayed formulations were measured using DSC on the powders' first heating to capture the morphology of the samples after spray drying. The melting point of L-leucine (286°C) is well above the degradation point of the samples, as well as L-leucine's own sublimation point, so this thermal transition was not observed in the DSC scans. For the ERL simul-sprays, multiple thermal events were observed. First, a weak glass transition temperature was observed with an onset of 34-37°C, characteristic of amorphous ERL. Second, a broad T_g was observed beginning at ~120°C, which matches that previously measured for the BEV formulation (1). Finally, a melt peak at ~165°C was observed. Comparing with the heat of fusion for pure crystalline ERL, the melt peak indicated that 78% of the ERL in the ERL 1:1 formulation was crystalline and 86% of the ERL in the ERL 1:2 formulation. This is in good agreement with the qualitative observations from the PXRD data.

The glass transition temperature of pure amorphous PTX was reported as 152°C [Liggins et al]. For all PTX simul-spray formulations, two separate glass transition temperatures were observed: one characteristic of the BEV/trehalose phase at 118°C onset, and a second at 150°C from the amorphous PTX. The relative magnitudes (delta C_P) of the two transitions were proportional to the simul-spray ratios.

For the CP simul-sprays, a glass transition temperature was observed starting at ~110°C, characteristic of the amorphous CP/trehalose phase. When the 10/70/20 CP trehalose/L-leucine formulation was spray dried by itself, an onset temperature of 109°C and midpoint of 113°C was observed, in good agreement with these results. The broad transition of the BEV/trehalose phase is present between ~118-140°C, but could not be quantified due to the overlap in its onset (118°C) with the end of the CP transition. The delta C_P of the CP transition was approximately proportional to the mass of CP powder in the simul-spray. An example thermogram for each of the CP formulations is shown in SI Figure E. The melting point of CP is well above the degradation point of other components of the formulations, and was therefore not measured by DSC.

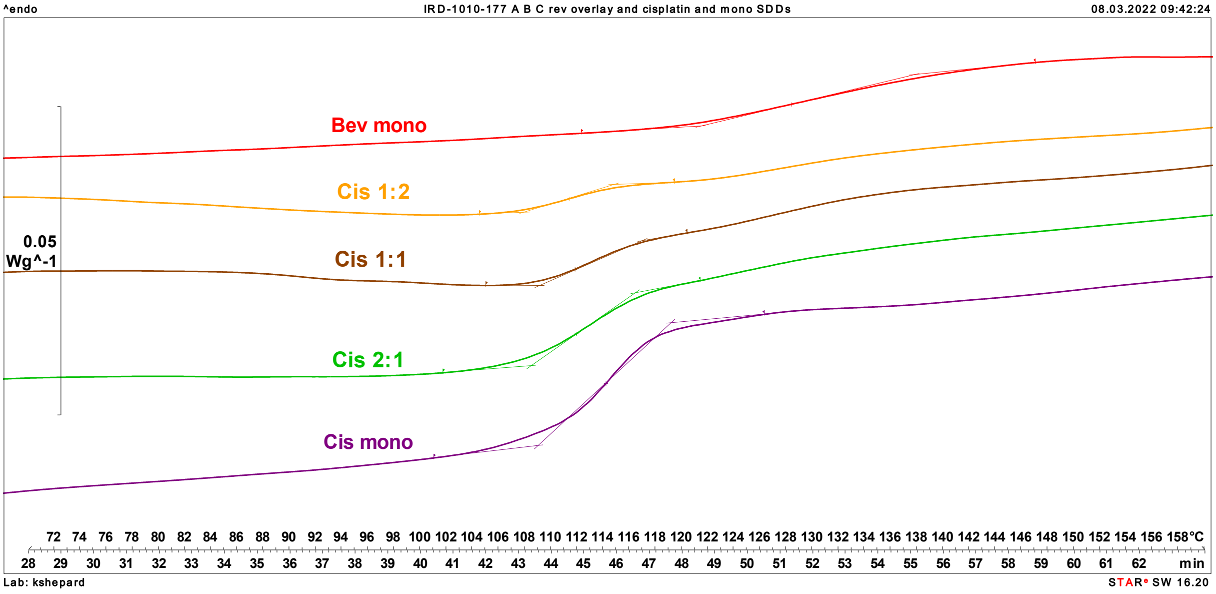

SI Figure E, DSC thermograms for CP formulations and controls.

Drug Concentration

BEV/CP simul-spray formulations were quantitated using the zero-intercept mode through Pion's Au PRO software. This technique compares the 2nd derivative of the absorbance spectra for each component (taken separately), and determines if a wavelength(s) exists at which component A can be quantitated without interference from component B, where the 2nd derivative spectrum of component B crosses the x-axis. If a suitable ZIM point(s) is found for each active, quantitation of 2 components in a single solution is possible, without separation as is achieved by HPLC. For the case of BEV/CP, BEV was quantified using the averaged standard curve from the 2nd derivative absorbance at 2 wavelengths: 282.01 and 298.11nm, as shown in SI Figure F. A ZIM point to quantitate CP is found around 292nm, but was not used as BEV has no absorbance beyond ~325nm. So CP was quantitated from the 2nd derivative standard curve in the 335 to 345nm range. An example spectrum of the test solution containing both BEV and CP is shown in SI Figure G.

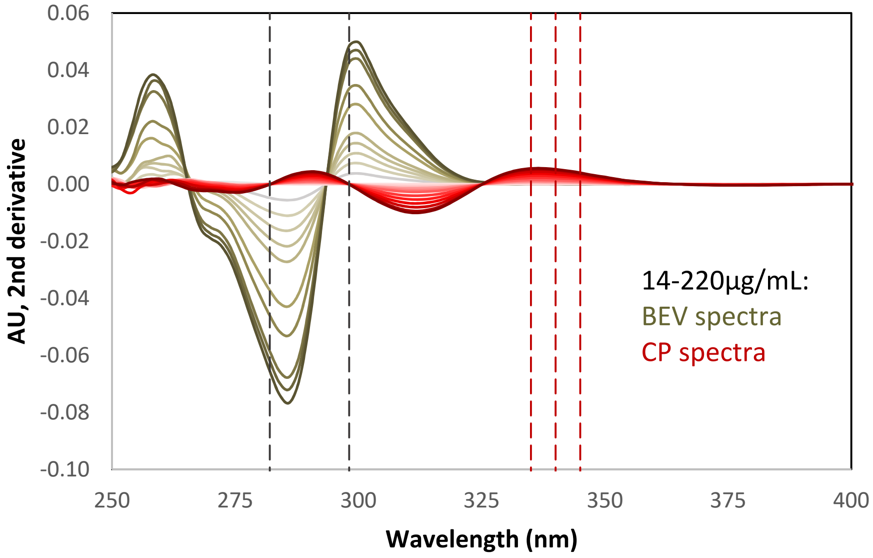

SI Figure F, 2nd derivative spectra for BEV and CP standard curves with dashed lines showing where each component was quantified.

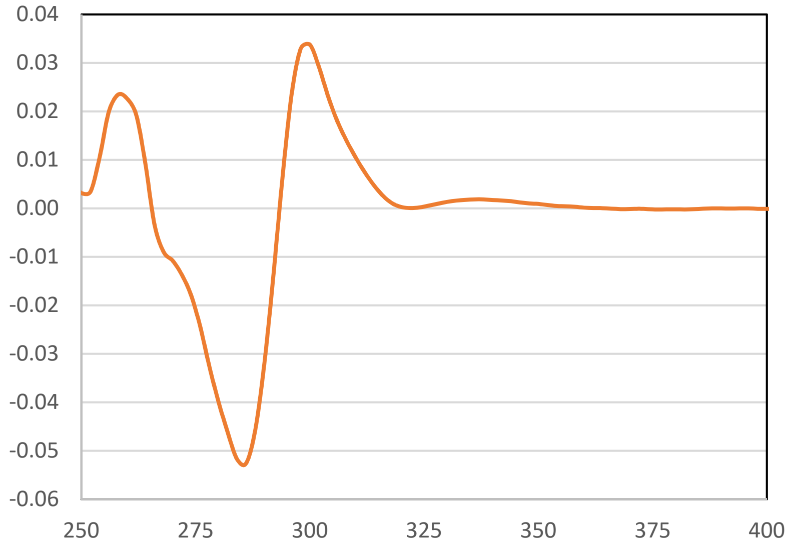

SI Figure G, 2nd derivative spectrum of a solution containing 2:1 BEV:CP.

Water content

To confirm the hypothesis about potency discrepancies resulting from water content differences in the individual formulations, all samples were equilibrated to the same ambient conditions overnight and then sampled for water content. The experimental versus predicted water content (based on the weighted averaged of the mono SDD water) is shown in SI Figure H against the 1:1 theoretical. Overall the water trends as predicted. The variation in water content for the individual formulations comprising the simul-spray SDDs along with the fact the samples were not uniformly equilibrated during potency sampling, makes true quantitation of the actives difficult, and robust sample prep and method development would be needed.

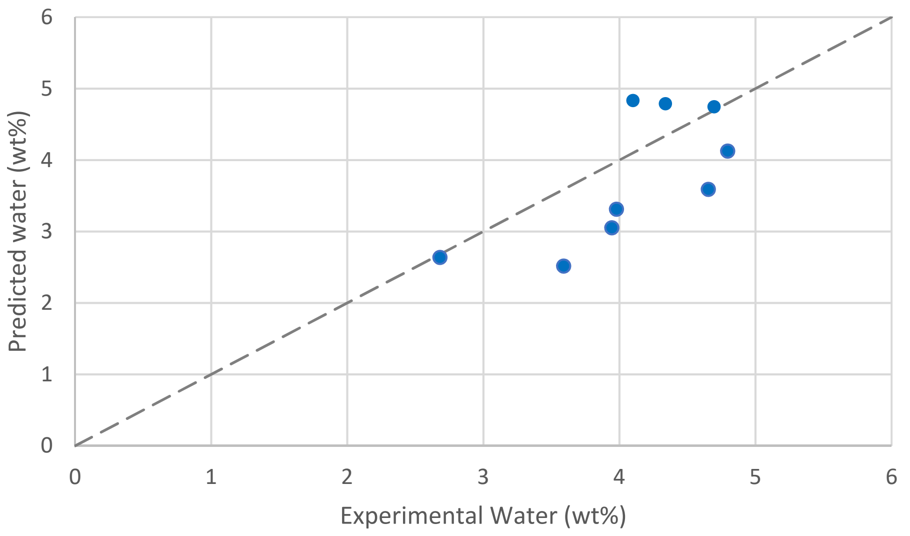

SI Figure H, predicted v. experimental water content for the 9 simul-spray formulations.

GC Headspace Instrument parameters

The parameters used for GC headspace analysis of residual solvent are in SI Table 1.

Injector temperature	180°C
Detector temperature	260°C
Oven program	40°C for 2 min, 50°C/min to 225°C, 0.5 min hold
Carrier gas flow	H_2, 38mL/min
FID air flow	400mL/min
FID Makeup gas flow	N_2, 30mL/min
Carrier flow rate	5mL/min
Split ratio	10:1

SI Table 1, Headspace sampler and instrument parameters for GC headspace analysis of residual solvent.

SI references

Shepard KB, Vodak DT, Kuehl PJ, Revelli D, Zhou Y, Pluntze AM, et al. Local Treatment of Non-small Cell Lung Cancer with a Spray-Dried Bevacizumab Formulation. AAPS PharmSciTech. 2021;22(7):230. doi: 10.1208/s12249-021-02095-7.

Liggins, R.T.; Hunter, W.; Burt, H.M. Solid-State Characterization of Paclitaxel. *J. Pharm. Sci.* **1997**, *86*, 1458–1463. https://doi.org/10.1021/js9605226
